# Immunohistochemical assays incorporating SP142 and 22C3 monoclonal antibodies for detection of PD-L1 expression in NSCLC patients with known status of *EGFR* and *ALK* genes

**DOI:** 10.18632/oncotarget.19724

**Published:** 2017-07-31

**Authors:** Paweł Krawczyk, Bożena Jarosz, Tomasz Kucharczyk, Anna Grenda, Katarzyna Reszka, Juliusz Pankowski, Kamila Wojas-Krawczyk, Marcin Nicoś, Justyna Szumiło, Tomasz Trojanowski, Janusz Milanowski

**Affiliations:** ^1^ Department of Pneumonology, Oncology and Allergology, Medical University of Lublin, Lublin, Poland; ^2^ Department of Neurosurgery and Pediatric Neurosurgery, Medical University of Lublin, Lublin, Poland; ^3^ Genetics and Immunology Laboratory, Genim LLC, Lublin, Poland; ^4^ Department of Pathology, Pulmonary Hospital, Zakopane, Poland; ^5^ Department of Pathomorphology, Medical University of Lublin, Lublin, Poland

**Keywords:** PD-L1, ALK, EGFR, non-small cell lung cancer

## Abstract

Different immunohistochemical (IHC) assays were approved for PD-L1 expression examination on tumor cells in qualification to immune-checkpoint inhibitors therapy in NSCLC patients. These assays have some similarities, but also very serious differences. We assessed 2 IHC tests for PD-L1 expression evaluation in NSCLC tumors with different pathological diagnoses and genetic abnormalities.

We enrolled 48 NSCLC patients (median age: 65 years) with known status of *EGFR* and *ALK* genes. We compared the effectiveness of PD-L1 expression examination of two IHC assays with 22C3 (Dako) and SP142 antibodies (Ventana). IHC tests were performed in resected tissue samples and in cellblocks from bronchoscopy biopsies (formalin-fixed paraffin-embedded). IHC staining was carried out on Dako Autostainer Link 48 and Ventana Benchmark GX.

The percentage of tumors with PD-L1 expression of ≥5% and ≥50% on tumor cells was significantly (p<0.05) higher in assay with 22C3 (66.7% and 45.8%) than with SP142 antibody (39.6% and 22.9%). The median percentage of tumor cells with PD-L1 expression was significantly (p<0.0001) higher in test with 22C3 than with SP142 antibody. Percentage of squamous cell carcinoma (SCC) patients with PD-L1 expression was significantly higher than of non-SCC patients. Large group of patients without PD-L1 expression on tumor cells was identified among patients with common *EGFR* mutations and *ALK* rearrangement.

Our results support that the highest PD-L1 expression on tumor cells occurs in SCC patients and in adenocarcinoma patients without common, druggable genetic abnormalities. The above mentioned results were clearly visible in IHC assay with 22C3 (strong cell staining).

## INTRODUCTION

Immunotherapies have been successfully used in the treatment of various neoplasms, including melanoma and non-small cell lung cancer (NSCLC). Three PD-1 (programmed death 1) or PD-L1 (programmed death ligand 1) inhibitors are approved in USA and/or in Europe for treatment of NSCLC patients. Nivolumab – a monoclonal anti-PD-1 antibody, is approved for advanced NSCLC patients after failure of first-line therapy. Pembrolizumab (monoclonal antibody against PD-1) can be used in advanced NSCLC patients in second- (tumor must contain at least 1% of PD-L1-positive tumor cells) or in first-line therapy (tumor must contain at least 50% of PD-L1-positive tumor cells). Atezolizumab is the first anti-PD-L1 antibody which was approved for patients with advanced NSCLC after failure of first-line therapy. Predictive factors for immunotherapy have not been fully explored. However, the association between effectiveness of anti-PD-1/anti-PD-L1 inhibitors and tumoral PD-L1 expression evaluated by immunohistochemistry (IHC) assays has been reported in clinical trials. Three different IHC assays, along with their corresponding drugs, were approved [1–7].

These IHC assays have some similarities, but also very serious differences. Each one was developed with unique primary antibody against PD-L1: 22C3 clone used in clinical trials with pembrolizumab, 28-8 clone studied in trials with nivolumab and SP142 clone used in trials with atezolizumab. IHC assays with 22C3 and 28-8 antibodies are manufactured by Dako and are optimized for the use with Autostainer Link 48 equipment, while SP142 was developed for Ventana BenchMark apparatus. The biggest problems with IHC assays created for PD-L1 expression examination are huge discrepancies in the way of results’ interpretation. Tumoral PD-L1 expression scoring is based on the assessment of the percentage of tumor cells expressing PD-L1. In IHC assay with 28-8 antibody, PD-L1 positive staining of tumor cells was assessed using three different thresholds (≥1%, ≥5% and ≥10% of tumor cells with PD-L1 expression). IHC assay with 22C3 clone considered two positive thresholds (≥1% and ≥50% of PD-L1-expressing tumor cells). An assessment of both tumor and tumor-infiltrating immune cells is required in IHC test with SP142 antibody. For tumor cells, three different cutoffs of positive PD-L1 staining have been considered in clinical trials: ≥1%, ≥5% and ≥50% of tumor cells with PD-L1 expression. The percentage area of tumor infiltrated by PD-L1 positive immune cells is assessed with cutoffs at ≥1%, ≥5% and ≥10% of the area with immune cells expressing PD-L1 [8–12].

Considering the extremely differential process of PD-L1 expression evaluation using different IHC assays and not fully established status of PD-L1 expression as a predictive factor for immunotherapy with PD-1 or PD-L1 inhibitors, it is necessary to examine differences in the expression of PD-L1 in patients with different clinical and molecular factors. The PD-L1 expression in patients with *EGFR* gene mutations and *ALK* gene rearrangement has been very poorly studied. Such patients rarely respond to treatment with immune-checkpoints inhibitors and should firstly be treated with molecularly targeted therapies [3, 7]. Our study estimated which of the IHC tests are the most useful in patients with different pathological diagnosis and with the studied genetic abnormalities.

## RESULTS

Patient demographics and clinical characteristics are summarized in Table 1.

**Table 1:** Clinical characteristic of patients

Gender	
Male, n(%)	20 (41.7)
Female, n(%)	28 (58.3)
**Median age ± SD (years)**	65 ± 7.6
**Smoking status**	
Smokers, n(%)	38 (79.2)
Non-smokers, n(%)	10 (20.8)
**Histopathology**	
Adenocarcinoma, n(%)	30 (62.5)
Squamous-cell carcinoma, n(%) (include 1 ADSC case)	15 (31.2)
NSCLC-NOS, n(%)	3 (6.3)
**Stage of disease**	
Early stages (I-IIIA), n(%)	34 (70.8)
Locally advanced stage (IIIB), n(%)	8 (16.7)
Advanced stage, n(%)	6 (12.5)
**Material submitted for analysis**	
Surgical material from primary tumor, n(%)	35 (72.9)
Surgical material from neurosurgery, n(%)	3 (6.3)
Intrabronchial forceps biopsy, n(%)	5 (10.4)
Cellblocks from EBUS/EUS-TBNA, n(%)	5 (10.4)
***EGFR* and *ALK* genes status**	
Patients with wild-type of both genes, n(%)	38 (79.2)
Patients with *EGFR* gene mutations, n(%)	7 (14.5)
Patients with *ALK* gene rearrangement, n(%)	3 (6.3)

**Figure 1:**
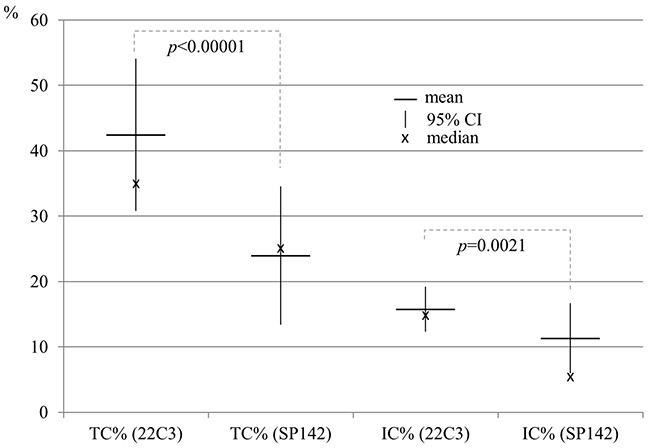
Percentage of tumor cells and percentage of tumor areas infiltrated with immune cells with expression of PD-L1 (% TPS) depending on antibody clones used in IHC technique

**Figure 2:**
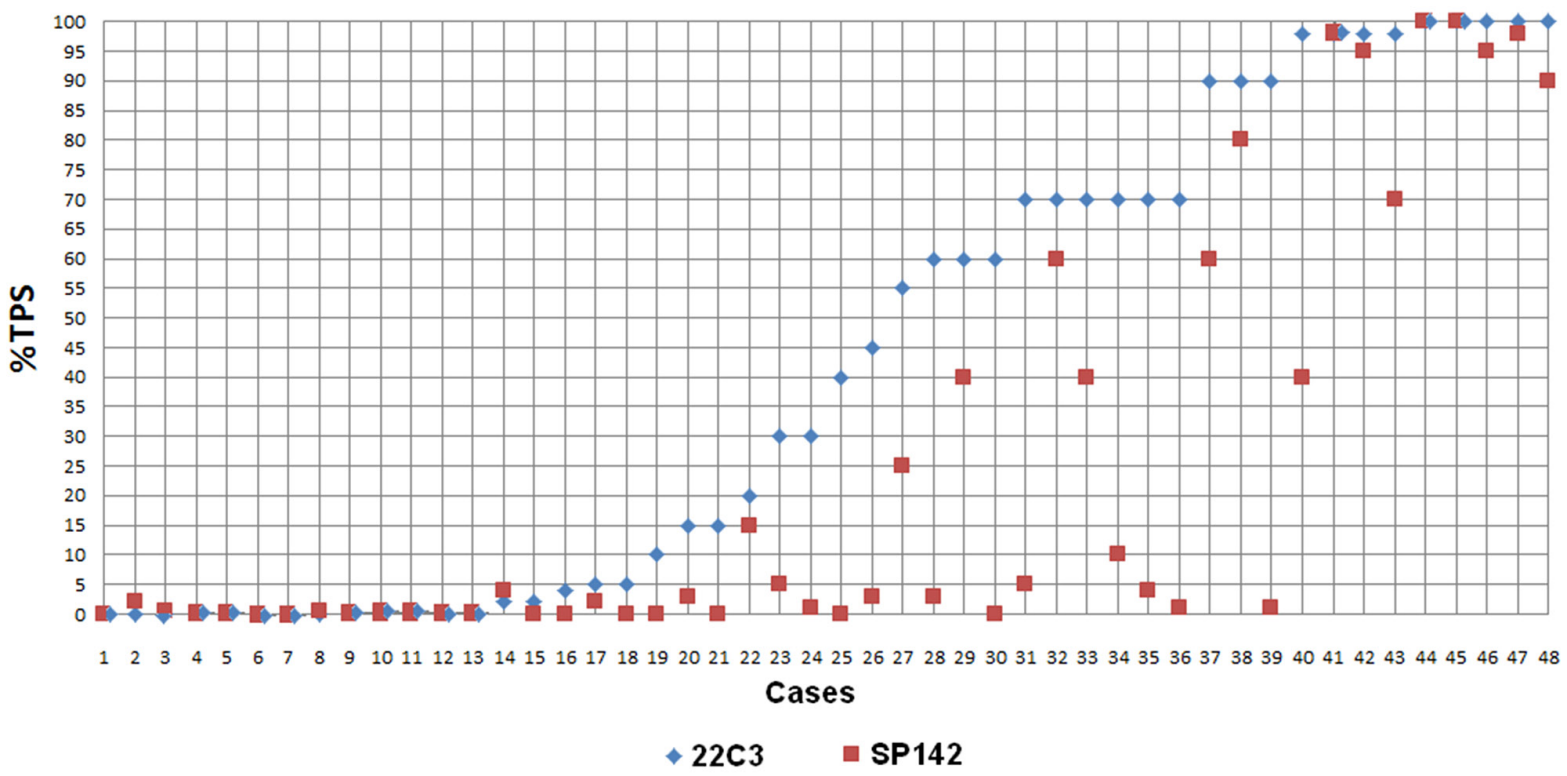
Comparison of percentage of tumor cells expressing PD-L1 stained with IHC assays using 22C3 and SP142 antibody clones Data points represent the mean score from observations by two pathologists for each assay, on each case.

**Figure 3:**
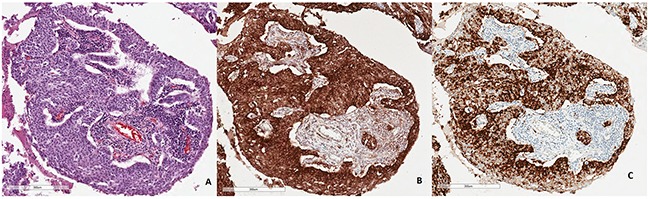
Representative IHC staining of PD-L1 on tumor cells using 22C3 and SP142 antibodies in the same NSCLC patients. Percentage of tumor cells with PD-L1 expression is 98% **(A)** Routine histopathological staining with hematoxylin and eosine. **(B)** Strong IHC staining of PD-L1 using 22C3 antibody. **(C)** Weaker IHC staining using SP142 antibody.

The percentage of tumors with PD-L1 expression on ≥1% of tumor cells was slightly higher in IHC reaction with 22C3 (72.9%) than with SP142 antibody (60.4%). The percentage of tumors with PD-L1 expression on ≥5% tumor cells was significantly (p<0.01) higher in IHC reaction with 22C3 (66.7%) than with SP142 antibody (39.6%). Similarly, the percentage of tumors with ≥50% of cells expressing PD-L1 was significantly (p<0.05) higher in IHC staining with 22C3 (45.8%) comparing to staining with SP142 antibody (22.9%). Moreover, the median percentage of tumor cells with PD-L1 expression detected by 22C3 antibody was significantly (p<0.0001) higher than percentage of these cells stained with SP142 antibody (Table 2, Figure 1 and 2). In most cases, weaker staining of tumor cells was observed in reaction with SP142, than with 22C3 antibody (Figure 3). The median percentage of tumor areas infiltrated with immune cells expressing PD-L1, detected by 22C3 antibody, was significantly (p=0.0021) higher than the median percentage of these areas in the assay with SP142 antibody (Figure 1).

**Table 2:** Percentage of cases with various PD-L1 expression on tumor cells visualized by immunohistochemistry method, using different clones of monoclonal antibodies against PD-L1 molecule

Monoclonal antibody clone used for slide staining	<1 % TPS	≥1 % TPS	<5% TPS	≥5% TPS	<50% TPS	≥50% TPS
**22C3, n(%)**	13 of 48 (27.1)	35 of 48 (72.9)	16 of 48 (33.3)	32 of 48 (66.7)	26 of 48 (54.2)	22 of 48 (45.8)
**SP 142, n(%)**	19 of 48 (39.6)	29 of 48 (60.4)	29 of 48 (60.4)	19 of 48 (39.6)	37 of 48 (77.1)	11 of 48 (22.9)
**Chi-square**:	1.688		7.069		5.587	
***p*-value**:	0.1939		0.0078		0.018	

Percentage of squamous cell carcinoma (SCC) patients with PD-L1 expression was generally higher than percentage of PD-L1-positive non-SCC patients. In IHC reaction with 22C3 antibody, 92.9% and 71.4% of SCC patients expressed PD-L1 on ≥1% and ≥50% of tumor cells, respectively. These values were significantly (p<0.05) higher than percentages of non-SCC patients who expressed PD-L1 on ≥1% and ≥50% of tumor cells (64.7% and 35.3%, respectively). In IHC reaction with SP142 antibody, percentages of SCC patients with PD-L1 expression on ≥5% and ≥50% of tumor cells (71.4% and 42.9%, respectively) were significantly higher (p<0.01 and p<0.05, respectively) compared to those of non-SCC patients (26.5% and 14.7%, respectively) (Table 3 and 4). In both IHC reactions, SCC patients had significantly (p<0.05) higher median percentage of PD-L1-positive tumor cells than patients with non-SCC (70% compared to 15% of PD-L1-positive tumor cells stained with 22C3 and 32.5% compared to 1% of PD-L1-positive tumor cells stained with SP142 antibody). In IHC reaction with 22C3 clone, the percentage of patients with PD-L1-expressing tumors on more than 5% of cells was significantly higher (p<0.05) in smokers than in non-smokers (Table 3). All patients with squamous cell carcinoma were smokers, and the majority of non-smokers were among patients with *EGFR* and *ALK* genes abnormalities. There were no significant differences between the median percentage of tumor areas infiltrated with immune cells in squamous and non-squamous cell lung cancer patients as well as in smoking or non-smoking patients.

**Table 3:** Percentage of cases with various PD-L1 expression on tumor cells visualized by immunohistochemistry method, using 22C3 monoclonal antibody in patients with different clinical characteristics

Clinical characteristic	<1 % TPS	≥1 % TPS	<5% TPS	≥5% TPS	<50% TPS	≥50% TPS
**Male, n(%)**	9 (36)	16 (64)	10 (40)	15 (60)	14 (56)	11 (44)
**Female, n(%)**	4 (17.4)	19 (82.6)	6 (26.1)	17 (73.9)	12 (52.2)	11 (47.8)
**Chi-square**:	2.101		1.043		0.071	
***p*-value**:	0.1472		0.3071		0.79	
**<67 years old, n(%)**	8 (30.8)	18 (69.2)	10 (38.5)	16 (61.5)	16 (61.5)	10 (38.5)
**≥67 years old, n(%)**	5 (22.7)	17 (77.3)	6 (27.3)	16 (82.7)	10 (45.4)	12 (54.6)
**Chi-square**:	0.39		0.671		1.242	
***p*-value**:	0.5323		0.4127		0.2651	
**Smokers, n(%)**	8 (21)	30 (79)	10 (26.3)	28 (73.7)	19 (50)	19 (50)
**Non-smokers, n(%)**	5 (50)	5 (50)	6 (60)	4 (40)	7 (70)	3 (30)
**Chi-square**:	3.359		4.042		1.276	
***p*-value**:	0.0668		0.0444		0.2586	
**SCC, n(%)**	1 (7.1)	13 (92.9)	2 (14.3)	12 (85.7)	4 (28.6)	10 (71.4)
**Non-SCC, n(%)**	12 (35.3)	22 (64.7)	14 (41.2)	20 (58.8)	22 (64.7)	12 (35.3)
**Chi-square**:	3.98		3.227		5.215	
***p*-value**:	0.0460		0.0724		0.0224	
**Early stages (I-IIIA)**, **n(%)**	7 (20.6)	27 (79.4)	9 (26.5)	25 (73.5)	19 (55.9)	15 (44.1)
**Advanced stages (IIIB-IV)**, **n(%)**	6 (42.9)	8 (57.1)	7 (50)	7 (50)	7 (50)	7 (50)
**Chi-square**:	2.49		2.471		0.138	
***p*-value**:	0.1146		0.116		0.7103	
**FFPE tissue, n(%)**	10 (23.3)	33 (76.7)	13 (30.2)	30 (79.8)	23 (53.5)	20 (46.5)
**Cellblock, n(%)**	3 (60)	2 (40)	3 (60)	2 (40)	3 (60)	2 (40)
**Chi-square**:	3.062		1.786		0.077	
***p*-value**:	0.08		0.1814		0.7814	
***EGFR* wt, n(%)**	10 (24.4)	31 (75.6)	13 (31.7)	28 (68.3)	22 (53.7)	19 (46.3)
***EGFR* mut, n(%)**	3 (42.9)	4 (57.1)	3 (42.9)	4 (57.1)	4 (57.1)	3 (42.9)
**Chi-square**:	1.033		0.334		0.029	
***p*-value**:	0.3095		0.5633		0.8648	
***ALK* rearrangement, n(%)**	3 (100)	0 (25)	3 (100)	0 (100)	3 (100)	0 (100)
No *ALK* rearrangement, n(%)	10 (22.7)	34 (77.3)	13 (29.5)	31 (70.5)	23 (52.3)	21 (47.7)
**Chi-square**:	8.381		6.209		2.588	
***p*-value**:	0.0038		0.0127		0.1077	

**Table 4:** Percentage of cases with various PD-L1 expression on tumor cells visualized by immunohistochemistry method, using SP142 monoclonal antibody in patients with different clinical characteristics

Clinical characteristic	<1 % TPS	≥1 % TPS	<5% TPS	≥5% TPS	<50% TPS	≥50% TPS
**Male, n(%)**	7 (35)	13 (65)	10 (50)	10 (50)	14 (70)	6 (30)
**Female, n(%)**	12 (42.9)	16 (57.1)	19 (67.9)	9 (32.1)	23 (82.1)	5 (17.9)
**Chi-square**:	0.301		1.556		0.974	
***p*-value**:	0.5833		0.2123		0.3237	
**<67 years old, n(%)**	12 (46.2)	14 (53.8)	17 (65.4)	9 (34.6)	19 (73.1)	7 (26.9)
**≥67 years old, n(%)**	7 (31.8)	15 (68.2)	12 (54.5)	10 (45.4)	18 (75)	4 (25)
**Chi-square**:	1.024		0.585		0.515	
***p*-value**:	0.3116		0.4444		0.4730	
**Smokers, n(%)**	12 (32.4)	25 (67.6)	22 (59.5)	15 (40.5)	32 (86.5	5 (13.5)
**Non-smokers, n(%)**	7 (63.6)	4 (36.4)	7 (63.6)	4 (36.4)	8 (72.7)	3 (27.3)
**Chi-square**:	3,452		0.062		1,156	
***p*-value**:	0.0631		0.8034		0.2823	
**SCC, n(%)**	3 (21.4)	11 (78.6)	4 (28.6)	10 (71.4)	8 (57.1)	6 (42.9)
**Non-SCC, n of 48 (%)**	16 (47.1)	18 (52.9)	25 (73.5)	9 (26.5)	29 (85.3)	5 (14.7)
**Chi-square**:	2.724		7.603		4.449	
***p*-value**:	0.0989		0.0058		0.0349	
**Early stages (I-IIIA), n(%)**	13 (38.2)	21 (61.8)	22 (64.7)	12 (35.3)	28 (82.4)	6 (17.6)
**Advanced stages (IIIB-IV), n(%)**	6 (42.9)	8 (57.7)	7 (50)	7 (50)	9 (64.3)	5 (35.7)
**Chi-square**:	0.089		0.897		1.832	
***p*-value**:	0.7655		0.3436		0.1759	
**FFPE tissue, n(%)**	16 (37.2)	27 (62.8)	26 (60.5)	17 (39.5)	32 (74.4)	11 (25.6)
**Cellblock, n(%)**	3 (60)	2 (40)	3 (60)	2 (40)	5 (100)	0 (0)
**Chi-square**:	0.973		0		1.659	
***p*-value**:	0.3239		1		0.1977	
***EGFR* wt, n(%)**	15 (36.6)	26 (63.4)	24 (58.5)	17 (41.5)	31 (75.6)	10 (24.4)
***EGFR* mut., n(%)**	4 (57.1)	3 (42.9)	5 (71.4)	2 (28.6)	6 (85.7)	1 (14.3)
**Chi-square**:	1.057		0.416		0.346	
***p*-value**:	0.3039		0.5189		0.5564	
**No *ALK* rearrangement, n(%)**	17 (38.6)	27 (61.4)	26 (59.1)	18 (40.9)	33 (75)	11 (25)
***ALK* rearrangement, n(%)**	2 (66.7)	1 (33.3)	3 (100)	0 (0)	3 (100)	0 (0)
**Chi-square**:	0.916		1.989		0.979	
***p*-value**:	0.3385		0.1584		0.3224	

The presence of PD-L1 expression on tumor cells and tumor-infiltrating immune cells did not depend on other demographic and clinical factors such as age, gender or stage of disease. However, PD-L1 expression tests in cellblocks were difficult for interpretation, and percentage of patients with PD-L1 expression detected in cellblocks was slightly lower than in FFPE tissue specimens (Table 3 and 4). Reliable assessment of PD-L1 expression on immune cells in cellblocks was impossible.

*EGFR* gene mutations were diagnosed in 7 adenocarcinoma patients (14,6% of all patients, 6 female, 1 male, median age: 64 years, 5 non-smokers, one current and one former smoker). Common *EGFR* gene mutations: exon 19 deletion and substitution p.Leu858Arg in exon 21 were found in 5 patients (wherein substitution p.Leu858Arg occurred only in one patient). Substitution p.Gly719X in exon 18 and p.Leu861Gln in exon 21 were diagnosed in single patients. Only one patient with common *EGFR* gene mutation (with deletion in exon 19) expressed PD-L1 on 70% (22C3 clone) and 4% (SP142 clone) of tumor cells in both IHC assays. In IHC assay using SP142 antibody, four patients with common *EGFR* gene mutations presented no expression of PD-L1, one of whom (with deletion in exon 19) showed PD-L1 expression on 10% of tumor cells (with 22C3 antibody). However, all patients with common *EGFR* gene mutations showed expression of PD-L1 on tumor-infiltrating immune cells (2%-20% of tumor area with PD-L1 expressing immune cells in both assays). Patients with rare *EGFR* gene mutations had strong PD-L1 expression. Female patient with p.Leu861Gln substitution showed PD-L1 expression on 40% of tumor cells and in immune cells (2% of tumor area) in both assays, while male patient with p.Gly719X substitution presented 90% (22C3) and 40% (SP142) of tumor cells with PD-L1 expression (in this patient 4% of tumor area showed immune cells with PD-L1 expression). Patient with p.Gly719X substitution, with strong PD-L1 expression, was treated with nivolumab in third-line therapy with high grade toxicity (fatigue) and rapid progression. In both IHC assays, the percentage of tumors with PD-L1 expression on ≥1%, ≥5% and ≥50% of tumor cells was similar in groups of patients with and without *EGFR* gene mutations (Table 3 and 4).

**Figure 4:**
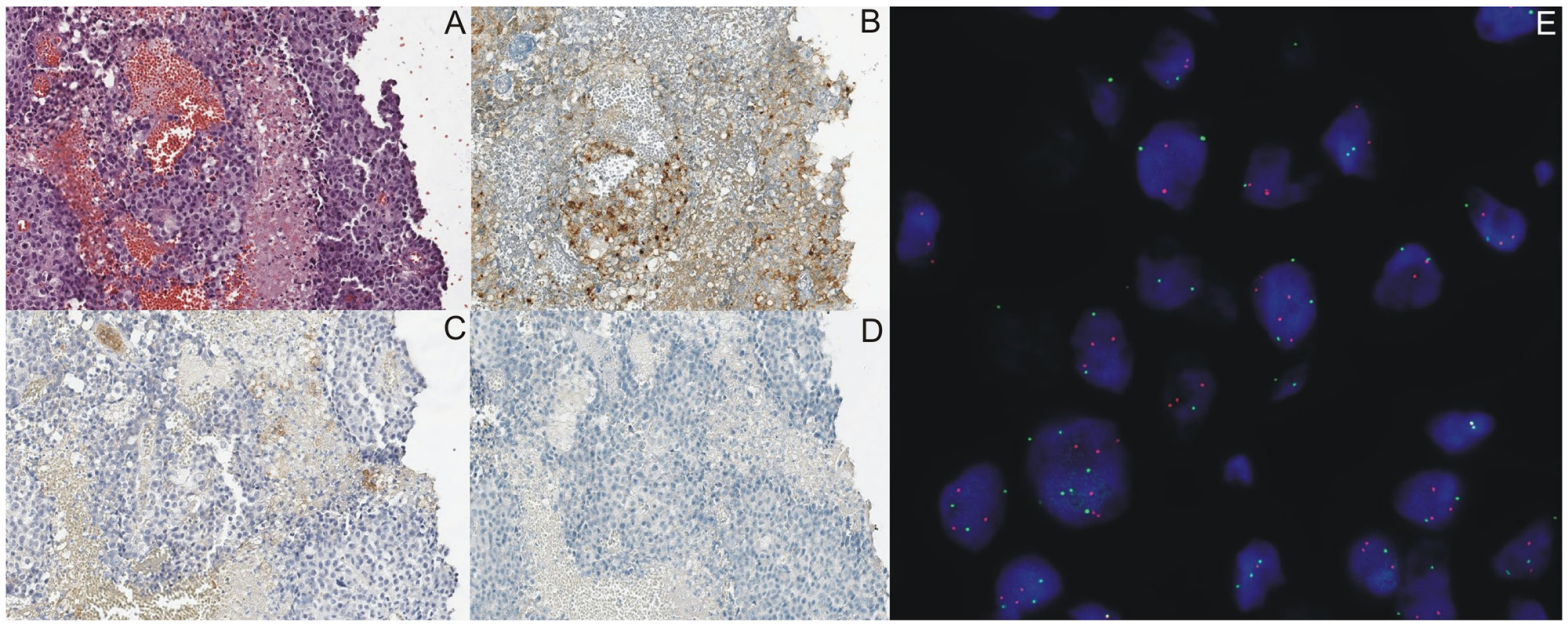
Representative IHC staining of ALK expression using D5F3 antibody and PD-L1 expression using 22C3 and SP142 antibodies on tumor cells in the same NSCLC patients with *ALK* gene rearrangement detected in FISH **(E)** and in RT-PCR method. **(A)** Routine histopathological staining with hematoxylin and eosine. **(B)** Percentage of tumor cells with abnormal ALK protein expression was 80%. **(C and D)** Lack of PD-L1 expression in IHC staining using 22C3 and SP142 antibodies.

Expression of abnormal ALK protein in IHC method was detected in 4 NSCLC patients. Using RT-PCR and FISH methods, *ALK* gene rearrangement was confirmed in three adenocarcinoma patients (6.25% of all patients, 2 female and one male, non-smoking patients) who expressed abnormal ALK protein on 60%-80% of tumor cells. *ALK* gene rearrangement was not confirmed in male patient with adenosquamous carcinoma, with abnormal ALK protein expression on only 5% of tumor cells. Patients with *ALK* gene rearrangement had no expression of PD-L1 on tumor cells in IHC assay with 22C3 antibody. One patient with *ALK* gene abnormality showed weak expression of PD-L1 on 2% of tumor cells in IHC staining with SP142 antibody (Table 3 and 4, Figure 4). This patient presented PD-L1 expression in 4-5% of the area infiltrated by PD-L1-expressing immune cells in both IHC assays.

## DISCUSSION

Our study confirmed that the PD-L1 expression examination results depend on many analytical and clinical factors. Primarily, the IHC assay with SP142 antibody results in a weaker staining of PD-L1 than the assay with 22C3 antibody. Therefore, negative results of PD-L1 expression on tumor cells are more frequently observed in staining using SP142 antibody than with 22C3 antibody. However, the recommendation to evaluate PD-L1 expression not only on tumor cells, but also on immune cells, are listed in the manufacturer's protocol of IHC assay using SP142. PD-L1 expression is often observed on tumor-infiltrating immune cells. Therefore, positive results of PD-L1 expression are more often achieved when using SP142 antibody than with 22C3.

Our observations are consistent with the results of the Blueprint IHC Assay Comparison Project. This study revealed that three IHC assays (with 22C3, 28-8 and SP263 antibody clones) were closely aligned on tumor cell staining, whereas the fourth IHC assay (with SP142 antibody clone) showed consistently fewer PD-L1 stained tumor cells. All of the assays demonstrated PD-L1 expression on immune cells, but with greater variability than on tumor cells. Authors concluded that differences in interpretation methods of IHC test results would lead to misclassification of PD-L1 status in some patients. However, this study was conducted on a small group of 38 patients. The correlation between PD-L1 expression and clinical, as well as genetic factors, was not analysed. Moreover, Hirsch and co-workers analysed PD-L1 expression only in large, surgically resected materials [12]. The expression of PD-L1 in a tumor is known to be very heterogeneous. McLaughlin and co-workers found a large variation in PD-L1 expression on tumor cells in different parts of the same tumor (from high expression to its complete absence) [13]. Ilie M et al. showed 48% of overall discordance rate between results of PD-L1 expression examination on tumor cells in surgical resected tissues and in biopsy specimens. Only 25% of surgical specimens and 74% of biopsy specimens did not express PD-L1 [14]. In our series, in 10 cases (among 48) expression of PD-L1 was analysed in intrabronchial biopsies or cell blocks from EBUS/EUS-TBNA. Therefore, these small samples could not be representative of the all tumor area.

Scheel et al. analysed PD-L1 expression in a large group of 436 genetically annotated NSCLC patients, using IHC assay with 5H1 antibody. Unfortunately, this antibody clone was not used for PD-L1 expression assessment in clinical trials with immune-checkpoint inhibitors. Moreover, the authors did not investigate abnormalities in *EGFR* and *ALK* genes, which are most relevant for patients’ qualification to molecularly targeted therapies. Sheel et al. showed no association between PD-L1 expression on tumor cells and clinical factors: pathological diagnosis, stage of disease, age and sex of patients. Whereas, PD-L1 expression in tumor cells was associated with presence of *KRAS* and *TP53* gene mutations as well as lack of mutations in *STK11* gene in adenocarcinoma patients [15].

Immunohistochemical assessment of PD-L1 expression on tumor and immune cells was performed and optimised during qualification of NSCLC patients to immune-checkpoint inhibitors therapy in several clinical trials. Effectiveness of different doses of pembrolizumab in patients with different status of PD-L1 expression on tumor cells was examined in Keynote-001 trial. This study used the assay with 22C3 antibody. Percentages of patients with PD-L1 expression on ≥1% or ≥50% of tumor cells, as well as percentage of patients without PD-L1 expression was similar in patients with squamous and nonsquamous NSCLC. Moreover, the *EGFR* gene status was not associated with changes in the proportion of patients with different PD-L1 expression on tumor cells. Surprisingly, all patients with *ALK* gene rearrangement demonstrated PD-L1 expression on tumor cells [4].

Effectiveness of nivolumab in comparison to docetaxel was evaluated in two clinical trials: CheckMate-057 for adenocarcinoma patients and CheckMate-017 for SCC patients. Expression of PD-L1 assessed by IHC reaction with 28-8 antibody on ≥1% of tumor cells was diagnosed in 54% of adenocarcinoma patients and in 52.8% of SCC patients. However, the percentage of SCC patients with PD-L1 expression on ≥10% of tumor cells was higher than of adenocarcinoma patients with the same PD-L1 expression cut-off. Patients with PD-L1 expression on ≥1% – <5% of tumor cells were predominant in adenocarcinoma patients’ group [1, 3].

The data on differences in PD-L1 expression on tumor and tumor-infiltrating immune cells, as well as on the effectiveness of atezolizumab, were available in descriptions of POPLAR and OAK clinical trials results. PD-L1 expression was assessed with SP142 antibody clone. Unfortunately, the results of these trials do not include information on PD-L1 expression in patients with different histology diagnosis and with different *EGFR* and *ALK* gene status [6, 7].

Rangachari D et al. analysed expression of PD-L1 using IHC assay with 22C3 antibody in 19 tumors with one of the genetic abnormalities: *EGFR* mutations, *ALK* or *ROS1* rearrangement. Significant majority of examined tumors showed no expression of PD-L1 or expression on <50% of tumor cells. Only one tumor had PD-L1 expression on ≥50% of tumor cells [16].

Our results support these findings, which indicate that highest PD-L1 expression on tumor cells occurs in SCC patients. The presence of *EGFR* gene mutations and, in particular, *ALK* gene rearrangement could be associated with the lack of PD-L1 expression on tumor cells. This could partly explain why patients with these genetic abnormalities are less responsive to treatment with immune-checkpoint inhibitors. The above mentioned results were clearly visible in IHC assay with 22C3 antibody, however not so obvious in IHC reaction with SP142 antibody. Moreover, this simple observation does not explain all the complex causes of low effectiveness of immunotherapy in patients with *EGFR* mutations and *ALK* rearrangement. PD-L1 expression by tumor cells is probably an active process protecting from an immune aggression. This immune aggression is less likely to occur against tumors with *EGFR* or *ALK* abnormalities since these tumors display less numerous mutations and thus less neo-antigens [17, 18].

We analysed PD-L1 expression in small populations of NSCLC patients and some results may be unreliable. However, we feel this study provided some important and significant insights for clinical practice. Firstly, our study population is greater than the number of IHC tested materials by Hirsh et al. (Blueprint Project) [12]. Furthermore, we included patients with known *EGFR* and *ALK* gene status, and such populations are rarely screened for PD-L1 expression. We confirmed not only statistically significant differences in PD-L1 expression in samples stained with 22C3 and SP142 antibody clones, but also in squamous and non-squamous cell lung cancer patients. On the other hand, we are aware that the number of patients with abnormalities in *EGFR* and *ALK* genes was too small to draw reliable conclusions about the differences in PD-L1 expression in patients with and without these abnormalities.

## MATERIALS AND METHODS

### Patients

We enrolled 48 NSCLC patients (median age: 65 ± 7.6 years). Tissue samples (formalin-fixed paraffin-embedded, FFPE) from surgically resected primary tumors, neurosurgically resected central nervous system (CNS) metastases and bronchoscopy biopsies (cellblocks) were available. Adenocarcinoma was diagnosed in 30 patients, squamous cell carcinoma in 15 and NSCLC NOS (not-otherwise specified) in 3 patients. All patients were radiotherapy and chemotherapy naive. The study was approved by the Ethics Committee of the Medical University of Lublin, Poland (No. KE-0254/169/2014).

### Methods

#### Immunohistochemistry

Immunohistochemical analyses of abnormal ALK and PD-L1 protein expression were carried out on paraffin embedded tissue cut into 3 μm sections and fixed on Thermo Scientific Superfrost Plus™ glass slides. All glass slides with tissue sections were preheated in 59°C on hotplate prior to IHC staining for at least 3 hours.

ALK protein IHC staining was carried out on Ventana Benchmark GX equipment, using CE-IVD approved anti-ALK Rabbit Monoclonal Primary Antibody (clone D5F3), utilizing OptiView Amplification Kit and OptiView DAB IHC Detection Kit as a detection system. Counterstaining, using hematoxylin (Ventana Medical System, Tucson, AZ, USA), was included in the staining protocol. Rabbit monoclonal negative control immunoglobulin (Ventana Medical System, Tucson, AZ, USA) was used as a negative control.

PD-L1 protein IHC staining was carried out using two different antibody clones – Ventana SP142 and Dako 22C3. The IHC staining procedure using Ventana antibody was carried out on Ventana Benchmark GX equipment, using CE-IVD approved Ventana PD-L1 (SP142) Assay, utilizing OptiView Amplification Kit and OptiView DAB IHC Detection Kit as a detection system. Counterstaining, using hematoxylin (Ventana Medical System, Tucson, AZ, USA), was included in the staining protocol. Rabbit monoclonal negative control immunoglobulin (Ventana Medical System, Tucson, AZ, USA) was used as a negative control.

The IHC staining procedure using Dako (Denmark) antibody was carried out on Dako Autostainer Link 48 equipment, utilizing CE-IVD approved PD-L1 IHC 22C3 PharmDx kit, using EnVision FLEX visualization system and counterstaining with hematoxylin, as a part of the staining protocol. Deparaffinization and antigen retrieval was carried out prior to the staining procedure on Dako PT Link equipment.

The cut of points for the assessment of cancer cell percentages with and without PD-L1 expression (<1%, 1-49% and ≥50% of tumor cells with PD-L1 expression) were adopted from the KEYNOTE-010 clinical trial, which compared the efficacy of pembrolizumab and docetaxel. In this study, the expression of PD-L1 was assessed with IHC assay using 22C3 antibody clone [5].

After staining all glass slides were washed and dehydrated in a series of two 96% ethanol and two xylene washing steps, and then covererslipped.

#### Fluorescence in situ hybridization (FISH)

All positive results of abnormal ALK protein expression obtained in IHC method were re-evaluated by FISH method to visualize the presence of *ALK* rearrangement using the Vysis ALK Break Apart FISH Probe Kit (Abbot Molecular, USA), Paraffin-Pretreatment IV and Post-Hybridization Wash Buffer Kit (Abbot Molecular, USA) and fluorescence microscope (Nikon Eclipse 55i, Japan). The localization and content of tumor cells in the specimens were examined with H&E staining in serially prepared slides. Way of interpretation of FISH results was in accordance to American Food and Drug Administration (FDA).

#### *Reverse transcription* polymerase chain reaction (*RT*-*PCR*)

The presence of abnormal mRNA containing sequence of *ALK-EML4* fusion gene was examined using reverse-transcription PCR method. mRNA was isolated from FFPE tissue samples using RNeasy FFPE Kit (Qiagen, Germany) according to manufacturer's protocol. For molecular analysis we used RT-PCR-based *EML4-ALK* Fusion Gene Detection Kit (Entrogen, USA) on Cobas Z 480 real-time PCR device (Roche Diagnostic, USA). The *EML4-ALK* Fusion Gene Detection Kit contains reagents for two step analysis that combine first-strand cDNA synthesis in reverse transcription and simultaneous amplification of mutant *ALK* and reference genes in a single reaction. The kit allows the detection of nine most common *EML4-ALK* fusion gene variants: 1: E13-A20; 2: E20-A20; 3a: E6-A20; 3b: E6-insA20; 4: E14-(−49)A20; 5a: E2-A20; 5b: E2-(+117)A20; 6: E13;(+69)A20; 7: E14-(13)A20. The kit detects 7 variants (1-3a/b,5a/b,6) in one reaction and 2 variants (4,7) in a second reaction. However, it does not distinguish between them. The negative control was determined with cDNA synthetized from mRNA isolated from peripheral blood leukocytes of healthy individuals and the positive control of the analysis was the reaction with control cDNA supplied with the assay by the manufacturer.

### Real-time PCR

Mutations analysis was performed when the presence of more than 10% of tumour cells was observed by a pathologist in H&E slides. Genomic DNA was extracted from the FFPE tumor tissue sections using a QIAamp DNA FFPE Tissue Kit (CE-IVD marked, Qiagen, Germany), according to the manufacturer's instructions. DNA concentration and quality was determined by spectrophotometry.

Mutations of *EGFR* gene (NM_005228.3) were tested using routine real-time PCR procedures and the EntroGen EGFR Mutations Analysis Kit (USA) on Cobas Z 480 real-time PCR device (Roche Diagnostics, USA). The following mutations in exons 18 to 21 were examined: p.Glu709Asp (c.2127A>C), p.Glu709Ala (c.2126A>C), p.Glu709Gly (c.2126A>G), p.Glu709Lys (c.2125G>A), p.Glu709Gln (c.2125G>C), p.Glu709Val (c.2126A>T), p.Gly719Ala (c.2156G>C), p.Gly719Ser (c.2155G>A), p.Gly719Cys (c.2155G>T); c.2235-2249 del 15, c.2235-2252>AAT del 18, c.2236-2253 del 18, c.2237-2251 del 15, c.2237-2254 del 18, c.2237-2255>T del 19, c.2236-2250 del 15, c.2238-2255 del 18, c.2238-2248>GC del 11, c.2238-2252>GCA del 15, c.2233-2247 del 15, c.2234-2248 del 15, c.2235-2246 del 12, c.2235-2248>AATTC, c.2235-2251>AATTC, c.2235-2252>AAT, c.2235-2255>AAT, c.2236-2248>AGAC, c.2236-2248>CAAC, c.2236-2256 del 21, c.2237-2252>T, c.2239-2247 del 9, c.2239-2256 del 18, c.2239-2248>C del 10, c.2239-2258>CA del 20, c.2240-2251 del 12, c.2240-2257 del 18, c.2240-2254 del 15, c.2239-2251>C del 13, c.2237-2253>TC, c.2237-2253>TTCCT, c.2237-2253>TTGCT, c.2237-2256>TC, c.2237-2256>TT, c.2237-2257>TCT, c.2238-2252 del 15, c.2239-2252>CA, c.2239-2253 del 15, c.2239-2256>CAA, c.2239-2257>T, c.2239-2262 del 24, c.2246-2260 del 15, c.2248-2273>CC, c.2252-2275 del 24, c.2252-2276>A, c.2252-2277>AT, c.2253-2276 del 24, c.2254-2277 del 24, p.Thr790Met (c.2369C>T), p.Ser768Ile (c.2303G>T), c.2307-2308 ins GCCAGCGTG, c.2319-2320 ins CAC, c.2310-2311 ins GGT; p.Leu858Arg (c.2573T>G), p.Leu858Met (c.2572C>A), p.Leu861Gln (c.2582T>A), p.Leu861Arg (c.2582T>G). The mutations analysis has been carried out in relation to the amplification of positive and negative control tests provided by the manufacturer and according to the included protocol.

### Statistical analysis

Statistical analysis was performed using Statistica v.10. Associations between PD-L1 expression, occurrence of *ALK* and *EGFR* genes abnormalities, as well as clinical factors were examined using the Fisher Chi-square test. The Wilcoxon test was used for testing the differences in medians of percentage of tumor cells stained in IHC reaction with different antibody clones. The U–Mann Whitney test was used for testing equality of population medians among subgroups. *P* values below 0.05 were considered significant.

## References

[1] Brahmer J, Reckamp KL, Baas P, Crinò L, Eberhardt WE, Poddubskaya E, Antonia S, Pluzanski A, Vokes EE, Holgado E, Waterhouse D, Ready N, Gainor J (2015). Nivolumab versus docetaxel in advanced squamous-cell non-small-cell lung cancer. N Engl J Med.

[2] Rizvi NA, Mazières J, Planchard D, Stinchcombe TE, Dy GK, Antonia SJ, Horn L, Lena H, Minenza E, Mennecier B, Otterson GA, Campos LT, Gandara DR (2015). Activity and safety of nivolumab, an anti-PD-1 immune checkpoint inhibitor, for patients with advanced, refractory squamous non-small-cell lung cancer (CheckMate 063): a phase 2, single-arm trial. Lancet Oncol.

[3] Borghaei H, Paz-Ares L, Horn L, Spigel DR, Steins M, Ready NE, Chow LQ, Vokes EE, Felip E, Holgado E, Barlesi F, Kohlhäufl M, Arrieta O (2015). Nivolumab versus docetaxel in advanced nonsquamous non-small-cell lung cancer. N Engl J Med.

[4] Garon EB, Rizvi NA, Hui R, Leighl N, Balmanoukian AS, Eder JP, Patnaik A, Aggarwal C, Gubens M, Horn L, Carcereny E, Ahn MJ, Felip E, KEYNOTE-001 Investigators (2015). Pembrolizumab for the treatment of non-small-cell lung cancer. N Engl J Med.

[5] Herbst RS, Baas P, Kim DW, Felip E, Pérez-Gracia JL, Han JY, Molina J, Kim JH, Arvis CD, Ahn MJ, Majem M, Fidler MJ, de Castro G (2016). Pembrolizumab versus docetaxel for previously treated, PD-L1-positive, advanced non-small-cell lung cancer (KEYNOTE-010): a randomised controlled trial. Lancet.

[6] Fehrenbacher L, Spira A, Ballinger M, Kowanetz M, Vansteenkiste J, Mazieres J, Park K, Smith D, Artal-Cortes A, Lewanski C, Braiteh F, Waterkamp D, He P, POPLAR Study Group (2016). Atezolizumab versus docetaxel for patients with previously treated non-small-cell lung cancer (POPLAR): a multicentre, open-label, phase 2 randomised controlled trial. Lancet.

[7] Rittmeyer A, Barlesi F, Waterkamp D, Park K, Ciardiello F, von Pawel J, Gadgeel SM, Hida T, Kowalski DM, Dols MC, Cortinovis DL, Leach J, Polikoff J, OAK Study Group (2017). Atezolizumab versus docetaxel in patients with previously treated non-small-cell lung cancer (OAK): a phase 3, open-label, multicentre randomised controlled trial. Lancet.

[8] Kerr KM, Tsao MS, Nicholson AG, Yatabe Y, Wistuba II, Hirsch FR, on behalf of the IASLC Pathology Committee (2015). Programmed death-ligand 1 immunohistochemistry in lung cancer: in what state is this art?. J Thorac Oncol.

[9] Cagle PT, Bernicker EH (2015). Challenges to biomarker testing for PD-1/PD-L1 checkpoint inhibitors for lung cancer. Arch Pathol Lab Med.

[10] Kerr KM, Hirsch FR (2016). Programmed death ligand-1 immunohistochemistry: friend or foe?. Arch Pathol Lab Med.

[11] Kerr KM, Nicolson MC (2016). Non-small cell lung cancer, PD-L1, and the pathologist. Arch Pathol Lab Med.

[12] Hirsch FR, McElhinny A, Stanforth D, Ranger-Moore J, Jansson M, Kulangara K, Richardson W, Towne P, Hanks D, Vennapusa B, Mistry A, Kalamegham R, Averbuch S (2017). PD-L1 immunohistochemistry assays for lung cancer: results from phase 1 of the Blueprint PD-L1 IHC Assay Comparison Project. J Thorac Oncol.

[13] Ilie M, Long-Mira E, Bence C, Butori C, Lassalle S, Bouhlel L, Fazzalari L, Zahaf K, Lalvée S, Washetine K (2016). Comparative study of the PD-L1 status between surgically resected specimens and matched biopsies of NSCLC patients reveal major discordances: a potential issue for anti-PD-L1 therapeutic strategies. Ann Oncol.

[14] McLaughlin J, Han G, Schalper KA, Carvajal-Hausdorf D, Pelekanou V, Rehman J, Velcheti V, Herbst R, LoRusso P, Rimm DL (2016). Quantitative assessment of the heterogeneity of PD-L1 expression in non-small-cell lung cancer. JAMA Oncol.

[15] Scheel AH, Ansén S, Schultheis AM, Scheffler M, Fischer RN, Michels S, Hellmich M, George J, Zander T, Brockmann M, Stoelben E, Groen H, Timens W (2016). PD-L1 expression in non-small cell lung cancer: Correlations with genetic alterations. Oncoimmunology.

[16] Rangachari D, VanderLaan PA, Shea M, Le X, Huberman MS, Kobayashi SS, Costa DB (2017). Correlation between classic driver oncogene mutations in EGFR, ALK, or ROS1 and 22C3-PD-L1 ≥50% expression in lung adenocarcinoma. J Thorac Oncol.

[17] Rizvi NA, Hellmann MD, Snyder A, Kvistborg P, Makarov V, Havel JJ, Lee W, Yuan J, Wong P, Ho TS, Miller ML, Rekhtman N, Moreira AL (2015). Cancer immunology. Mutational landscape determines sensitivity to PD-1 blockade in non-small cell lung cancer. Science.

[18] Shariat SF, Gust KM (2017). Immune therapy meets precision medicine. Lancet.

